# *Shewanella algae*-induced relapsing peritoneal dialysis-associated peritonitis: a case report

**DOI:** 10.3389/fmed.2025.1636954

**Published:** 2025-11-03

**Authors:** Jiawei Tang, Nana Luo, Yiming Zhang, Chen Wang

**Affiliations:** ^1^Clinical Medical College, Jining Medical University, Jining, Shandong, China; ^2^Department of Nephrology, Affiliated Hospital of Jining Medical University, Jining, Shandong, China; ^3^Department of Blood Purification, Affiliated Hospital of Jining Medical University, Jining, Shandong, China

**Keywords:** *Shewanella algae*, peritoneal dialysis-associated peritonitis, biofilm, catheter infection, antibiotics, antibiotic lock therapy

## Abstract

This article presents a case report of relapsing peritoneal dialysis-associated peritonitis caused by *Shewanella algae*. The patient has experienced relapsing peritonitis, accompanied by abdominal pain and cloudy peritoneal dialysis fluid. Ascites culture revealed an infection with *Shewanella algae*. Treatment with high-concentration gentamicin sulfate via intra-catheter antibiotic instillation led to complete resolution of the infection, with no recurrence observed. *Shewanella algae*-induced peritoneal dialysis-related peritonitis is rare, and its relapsing nature may be attributed to bacterial biofilm formation within the catheter. High-concentration antibiotic lock therapy demonstrated efficacy in eradicating the infection. This report explores the efficacy and application value of antibiotic lock therapy in managing relapsing *Shewanella algae*-associated peritoneal dialysis-related peritonitis.

## Introduction

This case report documents relapsing peritoneal dialysis-associated peritonitis (PDAP). According to guidelines issued by the International Society for Peritoneal Dialysis (ISPD), relapsing PDAP is explicitly defined as an episode that occurs within 4 weeks of completing antibiotic therapy for a prior episode and meets at least one of the following criteria: both episodes caused by the same organism; either episode culture-negative; or both episodes lacking definitive pathogen identification (1). The causative pathogen, *Shewanella algae*, is a gram-negative bacillus predominantly inhabiting marine environments. It can cause intra-abdominal infections, typically associated with seafood consumption or exposure to coastal waters (2, 3). Currently, PDAP caused by *S. algae* remains exceptionally rare, and its resistance mechanisms remain incompletely elucidated. In this case report, we implemented a novel high-concentration antibiotic lock therapy (ALT) regimen, exploring innovative therapeutic alternatives for recurrent PDAP.

## Case presentation

A 37-year-old male patient was admitted on September 27, 2024, presenting with “abdominal pain for 1 day after over 3 years of regular peritoneal dialysis.” The patient was diagnosed with chronic kidney disease stage 5 (uremia) 3 years ago and underwent peritoneal dialysis catheter insertion on November 4, 2021. The patient had been on automated peritoneal dialysis (APD) for 3 years. Nocturnal APD: 1.5% peritoneal dialysis solution 10,000 mL × 1 bag/night, delivered over 4 cycles in 11 h. Daytime: 2.5% solution 2,000 mL × 1 exchange/day (manual dwell).

The patient developed abdominal pain 1 day before admission without identifiable triggers, characterized by intermittent paroxysmal episodes, which later resolved. On the morning of admission, the peritoneal dialysis fluid was turbid and pale yellow; the patient denied concurrent pain, nausea, vomiting, or fever. This patient has a previous history of renal anemia, chronic heart failure, hypertensive disease, metabolic acidosis, osteonecrosis of the femoral head, renal cysts, and nephrolithiasis. Physical examination: body temperature 36.7 °C, heart rate 122 beats per minute, respiration 20 beats per minute, blood pressure 94/77 mmHg. No evident bleeding or exudation was observed in the outer tube of the peritoneal dialysis catheter. The abdomen was soft, with mild tenderness but without rebound tenderness. Positive shifting dullness was noted, and there was no edema in the bilateral lower extremities. After admission, the patient underwent a series of relevant auxiliary examinations. The ascitic fluid analysis revealed white blood cells at 2,533 × 10∧6/L, with 87% polymorphonuclear cells and 13% monocytes. Bacterial smear tests of the ascites did not detect any bacteria, fungi, or acid-fast bacilli. Blood routine examination combined with C-reactive protein (CRP) testing showed a white blood cell count of 7.42 × 10∧9/L, lymphocyte percentage of 15.90%, neutrophil percentage of 77.20%, and CRP level of 30.6 mg/L. Additionally, procalcitonin was measured at 1.33 ng/ml, and interleukin-6 was recorded at 360.00 pg/ml (Table 1). After admission, ascites were collected for culture, identification, and drug sensitivity testing. Given the patient's history of PDAP in August 2024, which was caused by *Escherichia coli* and successfully treated with intraperitoneal meropenem and vancomycin, an empirical regimen was initiated upon this admission. This included once-daily intraperitoneal meropenem (1 g) and vancomycin (1 g), concurrent with oral fluconazole (100 mg) once daily for fungal prophylaxis. Three days later, the patient experienced relief from abdominal pain, with a concomitant decrease in ascitic white blood cell count to 82 × 106/L, and the ascites culture results for the patient identified *Shewanella algae*. Despite a thorough investigation of potential risk factors, no recent history of exposure to coastal or freshwater environments, fish, or seafood handlers was reported. Antimicrobial susceptibility testing indicated that the isolate was susceptible to the following agents: ceftazidime, cefepime, ciprofloxacin, levofloxacin, doxycycline, and tigecycline (Table 2). Considering that the white blood cell counts in the patient's ascites remained elevated, the treatment regimen was adjusted based on the drug sensitivity results. Specifically, once-daily intraperitoneal administration of ceftazidime (1 g) and cefazolin sodium (1 g) were administered for 14 days (Table 3). The patient reported no abdominal pain during this period. Follow-up re-examination of the ascites revealed a reduced white blood cell count of 3 × 10∧6/L, and the ascites appeared clear.

**Table 1:** Laboratory test results.

**Variable**	**Reference range**	**The first hospitalization**	**Discharge from hospital**	**The second hospitalization**	**Discharge from hospital**	**The third hospitalization**	**Discharge from hospital**
White blood cell (×10^9^/L)	3.5–9.5	7.42	7.22	10.16	5.53	7.62	5.92
Hemoglobin (g/dL)	130–175	128	116	110	107	114	106
Hematocrit (%)	40–50	36.9	35.3	33.2	31.7	33.9	31.4
Platelet (×10^9^/L)	120–350	345	335	234	203	285	258
**Differential count (%)**							
Neutrophils	40–75	77.2	61.9	86.5	58.7	79.2	69.4
Lymphocytes	20–50	15.9	24.4	9	30.2	13.7	20.5
Monocytes	3–10	5.5	9	4.4	8.1	5.5	5.7
Serum IL-6 Assay	0–3.4	360		265			
Hepatitis B and C virus		Negative				Negative	
Human immunodeficiency virus		Negative				Negative	
Alanine aminotransferase (U/L)	9–50	7.7		4.2		35.2	24.1
Aspartate transaminase (U/L)	15–40	19.4				39	18
Albumin (g/dL)	40–55	33.5		30.3	28.6	39.4	39.3
Blood urea nitrogen (mmol/l)	3.1–8	19.1				31.2	24.7
Creatinine (umol/l)	57–97	1,215.2				1,205.1	1,509.6
Sodium (mmol/L)	137–147	136	139.5	135.6	140.2	131	138
Potassium (mmol/L)	3.5–5.3	3.23	4.18	3.77	4.58	3.44	4.88
Hydrogen carbonate (mmol/L)	22–29	22	24.7	31.9	28.7	23.9	27.4
Calcium (mmol/l)	2.11–2.52	2.52	2.46	2.28		2.51	2.39
C-Reactive Protein (mg/L)	0–6	30.6		36.3	2.3	5.2	1.11
Procalcitonin (ng/mL)	0–0.05	1.33	0.968	47.1	2.3	0.791	
**Peritoneal dialysis effluent analysis**							
Color		Pale yellow	Transparent	Transparent	Transparent	Pale yellow	Transparent
Clarity		Turbid	Clear	Clear	Clear	Turbid	Clear
White blood cell (×10^6^/L)		2,553	7	361	5	3,900	7
Dialysate culture		*Shewanella algae*		*Shewanella algae*		*Shewanella algae*	
Dialysate fungal stain and culture		Negative		Negative		Negative	
Dialysate acid fasting smear and culture		Negative		Negative		Negative	
Blood culture						Negative	

**Table 2:** Antibiogram of the microbiological exam.

**Identification result:** ***Shewanella algae***		
**Antibiotic**	**Result**	**Sensitivity**
Ticarcillin/Clavulanic Acid	≤8	S
Ceftazidime	0.5	S
Cefoperazone/Sulbactam	≤8	S
Cefepime	≤0.12	S
Aztreonam	≤1	S
Imipenem	≥16	R
Meropenem	2	S
Amikacin	≤2	S
Tobramycin	≤1	S
Ciprofloxacin	≤0.25	S
Levofloxacin	≤0.12	S
Doxycycline	≤0.5	S
Minocycline	≤1	S
Tigecycline	≤0.5	S
Trimethoprim/Sulfamethoxazole	≤20	S
Piperacillin/Tazobactam	≤4	S

**Table 3:** Antibiotics used during peritonitis.

**Hospitalization**	**Time**	**Dosage regimen**
The first hospitalization	Day 1 to Day 4	Meropenem1 g + vancomycin1 g (ip daily), fluconazole 100 mg (po daily)
	Day 6 to Day 20	Ceftazidime 1 g + cefazolin sodium 1 g (ip daily), fluconazole 100 mg (po daily)
The second hospitalization	Day 1 to Day 2	Ceftazidime 1 g + cefazolin sodium 1 g (ip daily)
	Day 3 to Day 4	Ceftazidime 1 g + cefazolin sodium 1 g + meropenem1g (ip daily), fluconazole 100 mg (po daily)
	Day 5 to Day 7	Ceftazidime 1 g + meropenem 1g (ip daily), fluconazole 100 mg (po daily)
	Day 8 to Day 21	Ceftazidime 1 g (ip daily), fluconazole 100 mg (po daily)
The third hospitalization	Day 1 to Day 2	Ceftazidime 1 g + cefazolin sodium 1 g (ip daily)
	Day 3	Ceftazidime 1 g + cefazolin sodium 1 g (ip daily), fluconazole 100 mg (po daily)
	Day 4 to Day 10	2 ml of gentamicin (80 mg/mL) lock in Tenckhoff catheter every day, retaining it for 6 h, fluconazole 100 mg (po daily)

On October 9, 2024, the patient was readmitted due to “half-day abdominal pain accompanied by turbid peritoneal dialysis fluid.” Upon admission, ascitic fluid analysis was performed, revealing a white blood cell count of 361 × 10∧6/L (Table 1). The patient was initially treated with once-daily intraperitoneal administration of ceftazidime (1 g) and cefazolin sodium (1 g) administered for 2 days. A subsequent re-examination indicated an elevated white blood cell count in the ascites at 1,634 × 10∧6/L. Consequently, once-daily meropenem (1 g) was added for intraperitoneal administration of antibiotic therapy. On hospital day 3, the patient's peritoneal dialysis effluent white blood cell count decreased to 56 × 10∧6/L. In addition, oral fluconazole (100 mg) once daily prophylaxis was initiated. On the fourth day of hospitalization, the ascitic fluid culture is positive for *Shewanella algae*. Relapsing peritoneal dialysis-associated peritonitis is being considered. At this point, the white blood cell count in the ascites had decreased to 20 × 10∧6/L. Due to relapsing infection with the same pathogen, consider for relapsing peritoneal dialysis-associated peritonitis, clinical guidelines recommend removal of the peritoneal dialysis catheter. However, the patient refuses catheter removal and opts to continue peritoneal dialysis therapy. Adjusting once-daily intraperitoneal administration of antibiotic therapy with ceftazidime (1 g) and meropenem (1 g) for 3 days. During this period, the white blood cell count dropped to 7 × 106/L in the ascites, and the patient no longer experienced abdominal pain. As a result, meropenem was discontinued. Following 14 days of once-daily intraperitoneal administration of ceftazidime (1 g) therapy (Table 3), the patient remained asymptomatic, with a routine ascites re-examination showing a white blood cell count of 11 × 10∧6/L and clear ascites.

On November 29, 2024, the patient was readmitted due to “cloudy peritoneal dialysate for 1 day.” The peritoneal dialysis fluid was turbid and pale yellow. Ascitic fluid analysis revealed a white blood cell count of 3,900 × 10∧6/L and culture-confirmed relapsing *Shewanella algae* infection (Table 1). After 3 days of once-daily intraperitoneal administration of ceftazidime (1 g) and cefazolin sodium (1 g), the dialysate cleared, and the white blood cell count dropped to 14 × 106/L. oral fluconazole (100 mg) once daily was concurrently initiated for prophylactic antifungal coverage. Considering the patient's relapsing *S. algae*-associated peritoneal dialysis-related peritonitis, likely attributable to biofilm formation within the dialysis catheter, during the patient's daytime dwell period, we perform a daily antibiotic lock (Gentamicin 80 mg/mL, total 2 mL) to fill his catheter (Table 3). The lock solution was drained 6 h later when he performed the nocturnal APD exchange. There was dialysate in the peritoneal cavity during the antibiotic lock in the catheter. The patient was discharged after 1 week of treatment. During the 4-month follow-up period, no recurrence of infection was observed.

## Discussion

The *Shewanella* genus comprises oxidase-positive, facultatively anaerobic, gram-negative bacilli, first described by Derby and Hammer in 1931 (3). Among clinical specimens, *Shewanella algae* is the predominant species associated with human infections (4). *S. algae* can cause bacteremia, soft tissue infections, and intra-abdominal infections (5).

To date, only two cases of peritoneal dialysis-associated peritonitis caused by *S. algae* have been reported, with one fatal outcome and another case showing clinical improvement after intravenous gentamicin therapy without recurrence (6). A 2024 study on bacterial identification in peritoneal dialysis effluent (PDE) revealed that *Shewanella algae* was among the relatively abundant bacterial species identified in metagenomic analyses of 89 PDE samples, although it was not a dominant pathogen. Its presence correlates with catheter-associated infections, and further research linking it to confirmed cases of peritonitis is needed to determine its actual clinical incidence (7). A genomic study of *S. algae* strains isolated in Hainan, China, revealed that nearly all of the 62 analyzed strains carried resistance genes to quinolones, sulfonamides, β-lactams, aminoglycosides, and polypeptides (8). Additionally, genomic sequencing analysis has revealed that *Shewanella algae* harbors blaOXA, blaAmpC, and qnr genes, which confer intrinsic resistance to carbapenems and quinolones. Notably, the blaAmpC gene further reduces susceptibility to cephalosporins by encoding blaAmpC β-lactamase, thereby hydrolyzing third-generation cephalosporins (9, 10). However, clinical studies indicate that treatment with β-lactams, aminoglycosides, or quinolones generally achieves favorable outcomes in patients with infections at various sites (11).

Bacterial biofilms are ubiquitous microbial communities in moist environments and a major cause of infections associated with implantable medical devices (12). Most antibiotic susceptibility testing is performed on planktonic (free-floating) bacteria. However, within biofilms, a small subpopulation known as persister cells exhibits markedly increased tolerance to antibiotics, with resistance levels up to 1,000-fold higher than those of planktonic bacteria (13, 14). These persister cells can resume active growth once antibiotics are discontinued, leading to recurrent infections (15). These persister cells can resume active growth once antibiotics are discontinued, leading to recurrent infections (16). Biofilm formation provides bacteria with a stable microenvironment, enabling persistent colonization in the host (17). In *Shewanella* species, biofilms facilitate bacterial colonization on surfaces such as catheters (18), a common mechanism in catheter-related infections. Liu et al. investigated 18 peritoneal dialysis catheters removed due to recurrent peritonitis. Bacterial cultures and scanning electron microscopy (SEM) revealed biofilms in 16 catheters, with SEM confirming complete luminal coverage by bacterial biofilms and no coexisting microbial types (19). In this case, the patient's recurrent peritoneal dialysis-associated peritonitis likely stemmed from *Shewanella algae* biofilm colonization within the catheter. Standard antibiotic doses may have cleared superficially exposed active bacteria but failed to eradicate protected persister cells embedded in the biofilm matrix (16). These viable persister cells undergo phenotypic adaptations, allowing them to revert to metabolically active states post-antibiotic therapy, driving persistent or relapsing infections. This phenomenon may explain the discordance between the pathogen's apparent *in vitro* antibiotic susceptibility and the patient's recurrent clinical infections. To address biofilm-mediated resistance, localized delivery of high-concentration antimicrobial agents directly to the infected site (e.g., catheter lock therapy) may enhance biofilm penetration and eradicate protected bacterial subpopulations. This approach aligns with the successful outcome observed in this case following gentamicin catheter lock therapy.

According to the Chinese Guidelines for Dialysis Access, patients with peritoneal dialysis-related peritonitis accompanied by catheter exit-site or tunnel infections are typically advised to undergo peritoneal dialysis catheter removal, followed by immediate transition to hemodialysis (20). However, in this case, the patient's ascitic fluid white blood cell count decreased after antibiotic therapy, and the patient strongly insisted on continuing peritoneal dialysis. This prompted consideration of antibiotic lock therapy, a strategy commonly used in hemodialysis patients with central venous catheters (CVCs). Studies indicate that bacterial colonization on implanted catheters can occur within 24 h, with biofilm formation in catheter lumens observed after >30 days of implantation (12). Antimicrobial lock solutions, containing antibiotic-heparin or antibiotic-citrate combinations, have proven effective in reducing biofilm formation and preventing catheter-related infections during interdialytic intervals (21). ALT involves instilling high-concentration antibiotic solutions (often combined with anticoagulants) into the catheter lumen when not in use, effectively disinfecting the catheter, treating infections, and avoiding catheter removal (22, 23). This approach is particularly valuable for catheter-dependent patients at high risk of recurrent catheter-related bloodstream infections (CRBSI). Guidelines recommend evidence-based antibiotic lock solutions for high-risk CRBSI cases, such as cefotaxime, gentamicin, or trimethoprim-sulfamethoxazole (20). Considering that the patient's multiple intraperitoneal injections of cephalosporin for the treatment of peritonitis have all recurred. Therefore, the patient was given high-concentration gentamicin sulfate catheter sealing treatment without removing the peritoneal dialysis catheter. After the treatment, the peritonitis of the patient did not recur, and the sufficiency of peritoneal dialysis met the standard.

Under high-concentration antibiotic exposure, all bacterial populations in planktonic or biofilm states demonstrate negative net growth, where antibiotic-induced mortality exceeds replication rates. Although biofilm biomass may decline temporarily, residual biofilm structures can persist until antibiotic degradation, subsequently resuming slow repopulation. Notably, emerging evidence suggests that elevated antibiotic concentrations might paradoxically accelerate the evolution of bacterial resistance (14). Furthermore, indiscriminate use of ALT in CRBSI has been associated with increased antimicrobial resistance (24). These findings emphasize the critical importance of pharmacokinetic optimization, particularly regarding antibiotic half-life and degradation kinetics, when designing ALT regimens for peritonitis management.

This article focuses on the diagnosis and management of recurrent peritonitis caused by *Shewanella algae* infection. Additionally, it highlights the feasibility of high-concentration antibiotic therapy for recurrent peritonitis or catheter-related infections in peritoneal dialysis patients. However, further research is required to optimize antibiotic selection and dosing protocols. Ultimately, catheter removal remains the definitive treatment strategy for refractory peritonitis associated with catheter infections.

## Data Availability

The original contributions presented in the study are included in the article/supplementary material, further inquiries can be directed to the corresponding authors.
